# Tuberculosis in Children and Adolescents: Progress and Perseverance

**DOI:** 10.3390/pathogens11040392

**Published:** 2022-03-24

**Authors:** Stephen M. Graham, Ben J. Marais, Farhana Amanullah

**Affiliations:** 1 Department of Paediatrics and Murdoch Children’s Research Institute, University of Melbourne, Royal Children’s Hospital, Melbourne, VIC 3052, Australia; 2 The Burnet Institute, Melbourne, VIC 3004, Australia; 3 Department of Paediatrics and Child Health, The Children’s Hospital at Westmead, The University of Sydney, Westmead, NSW 2145, Australia; ben.marais@health.nsw.gov.au; 4 Department of Paediatrics, The Indus Hospital and Health Network, The Aga Khan University Hospital, Karachi 75500, Pakistan; farhana.amanullah@tih.org.pk

Although it is an ancient pathogen, tuberculosis (TB) remains a major infectious cause of death globally, transiently displaced by COVID-19 [1]. Since the development of antibiotics to treat TB more than half a century ago, public health has largely focused on detecting and successfully treating the most infectious patients in communities. Only recently have other vulnerable populations received due attention [2,3]; moreover, despite the far greater inclusion of children in policy development, advocacy, research, and programmatic implementation today, many challenges remain. Given that the COVID-19 pandemic has stalled and even reversed progress in global TB control [1], the publication of this Special Issue in *Pathogens* is extremely timely.

Children and adolescents in high-incidence settings continue to experience a huge burden of TB, despite it being treatable and preventable [4,5]. Infants and young children are at high risk of severe and disseminated disease associated with death or long-term disability, while adolescents commonly develop adult-type pulmonary TB, which is highly infectious and contributes to ongoing transmission within communities. The major challenges include reducing missed opportunities for TB prevention, of which there have been many, and increasing case detection, especially in young children [1]. Identifying those with drug-resistant TB is essential for providing appropriate treatment. Historically, management guidelines for child TB were largely informed by evidence extrapolated from adult populations and by expert opinions based on clinical experience in a limited number of settings [6]. Consolidated guidelines were launched on World TB Day 2022 by the World Health Organization (WHO); these represent a major milestone on the road to improving TB prevention and care in vulnerable young children, and in addressing the unique needs of adolescents. These WHO guidelines include new and updated recommendations based on the best current evidence and are accompanied by an operational handbook that aims to respond to the needs of all relevant sub-populations [7,8]. 

In the context of this recent progress, this Special Issue of *Pathogens* is dedicated to child and adolescent TB. It combines evidence updates with critical consideration of persistent policy–practice gaps and covers a wide range of issues. The content covers pragmatic approaches to prevention and advances, as well as opportunities to improve case detection, clinical and laboratory diagnosis, and treatment outcomes. The need for national leadership, integration of services, community engagement, enhanced data collection, and reporting, as well as greater investment in research and implementation, are highlighted. The authors represent global and national leaders in epidemiology, clinical management, research, policy, and advocacy. Additionally, many are young and emerging researchers. Many of the authors contributed to formal evidence reviews to inform the development of new recommendations and to consolidate updated policy guidelines, together with a detailed operational handbook [7,8]. 

Expertise in the management of TB in children is slowly strengthening in endemic countries, as evidenced by increasing involvement in TB research and collaboration, and by increasing recognition from National TB programs and pediatric societies [9,10]. However, efforts are still, often, at an early gestational stage, lacking ‘critical mass’ and broad program support in many endemic settings. Even in well-resourced countries, challenges remain to improving the delivery of comprehensive TB care to children and adolescents, requiring persistent attention and advocacy [11]. A multisectoral approach is critical to close the wide policy–practice gaps that hamper service delivery. Specifically, this requires close collaboration between national TB programs and maternal, child and adolescent health services [12,13].

We still rely heavily on estimates of global or national disease burden rather than actual data due to major gaps in case detection, notification and vital registration [14]. Only recently has there been a better appreciation of the unique needs and challenges of adolescents with TB [15,16]. Improving detection, treatment and prevention across the full age spectrum, from infants to young adults, requires age-disaggregated data, to monitor progress and persistent gaps in service delivery. Case notification data for child and adolescent TB have traditionally been reported within three age bands: 0–4, 5–14 and 15–24 years. With the increasing uptake of electronic data systems, there is now a transition to reporting case notifications and treatment outcomes in more informative 5-year age bands: 0–4, 5–9, 10–14, 15–19 and 20–24 years. This will improve the knowledge and understanding of global and national TB burdens in specific high-risk groups, such as young children (0–4 years) and older adolescents (15–19 years), at a more granular level. In addition, it will help to focus attention on particular clinical presentations and diagnostic challenges, for example, of children less than 10 years old who are generally unable to expectorate, and who develop less-specific radiological abnormalities than adolescents (10–19 years) [17]. Important age-related differences are reflected in clinical and radiological presentation, as are sampling techniques and the yield of bacteriological confirmation from various samples [17,18,19]. As clinical diagnosis is still required for most children with TB, accurate point-of-care tests remain a priority for development [10,20].

Given the major case detection gap especially in young children, the focus on strengthening clinical, radiological and laboratory diagnoses at all levels of the health system is a major step forward. Further, there is important progress towards improved treatment adherence and outcomes. Shorter regimens are now recommended for the treatment of non-severe disease and infection resulting from drug-susceptible TB [7,21,22]. An updated guide has been developed by The Union to support and strengthen chest X-ray interpretation for treatment decisions [23]. New recommendations also provide opportunities to improve the outcomes and safety for treatment of the most challenging forms of TB, such as TB meningitis and multidrug-resistant TB [24,25]. Child-friendly formulations of key second-line drugs are increasingly available [8,25]. 

Tuberculosis is a disease characterized by inequity. It is most prevalent in poor and vulnerable communities, directly contributing to the further impoverishment and increased vulnerability of those populations. There are now universal recommendations and important opportunities to provide timely, effective, and safe TB-preventive treatment to all child and adolescent contacts—including those living with HIV—in an integrated, community-based context [8,22]. Community engagement and support with education, de-stigmatization, and ensuring access to care for those affected is critical. Most children with TB are not treated at specialized TB services at a tertiary level in an urban setting, but rather, at general primary and secondary child health-care services. Therefore, the decentralization of services is key to improving the coverage of detection, treatment, and prevention [26]. The integration of services, such as comprehensive TB services for children and adolescents living with HIV, is clearly critical [27]. Integration also extends to families and households. While coverage of TPT for people living with HIV has been well integrated within HIV services, the coverage of TPT for eligible child contacts is incredibly low [1,28]. There is now increasing evidence from TB-endemic settings that comprehensive, community-based household contact screening and management can be successfully decentralized and integrated to increase case detection and treatment, as well as prevention [9,26,28].

This Special Issue celebrates and complements the WHO’s updated, consolidated guidelines and accompanying operational handbook [7,8]. We are grateful for the many excellent contributions that provide updated reviews of the evidence and ongoing challenges. We hope that this Special Issue will be a valuable resource as we emerge from the devastating impacts of the COVID-19 pandemic, with renewed focus and enthusiasm towards ending TB-related morbidity and mortality in children and adolescents.
